# Biased Random Walk With Restart on Multilayer Heterogeneous Networks for MiRNA–Disease Association Prediction

**DOI:** 10.3389/fgene.2021.720327

**Published:** 2021-08-10

**Authors:** Jia Qu, Chun-Chun Wang, Shu-Bin Cai, Wen-Di Zhao, Xiao-Long Cheng, Zhong Ming

**Affiliations:** ^1^School of Computer Science and Artificial Intelligence & Aliyun School of Big Data, Changzhou University, Changzhou, China; ^2^Information and Control Engineering, China University of Mining and Technology, Xuzhou, China; ^3^College of Computer Science and Software Engineering, Shenzhen University, Shenzhen, China

**Keywords:** microRNA, disease, association prediction, degree, biased random walk with restart

## Abstract

Numerous experiments have proved that microRNAs (miRNAs) could be used as diagnostic biomarkers for many complex diseases. Thus, it is conceivable that predicting the unobserved associations between miRNAs and diseases is extremely significant for the medical field. Here, based on heterogeneous networks built on the information of known miRNA–disease associations, miRNA function similarity, disease semantic similarity, and Gaussian interaction profile kernel similarity for miRNAs and diseases, we developed a computing model of biased random walk with restart on multilayer heterogeneous networks for miRNA–disease association prediction (BRWRMHMDA) through enforcing degree-based biased random walk with restart (BRWR). Assessment results reflected that an AUC of 0.8310 was gained in local leave-one-out cross-validation (LOOCV), which proved the calculation algorithm’s good performance. Besides, we carried out BRWRMHMDA to prioritize candidate miRNAs for esophageal neoplasms based on HMDD v2.0. We further prioritize candidate miRNAs for breast neoplasms based on HMDD v1.0. The local LOOCV results and performance analysis of the case study all showed that the proposed model has good and stable performance.

## Introduction

MicroRNA (miRNA) is a noncoding single-stranded RNA with a length of about 22 nucleotides and pervasive in both animals and plants (Axtell et al., 2011). MiRNAs play their regulator role through binding to imperfect complementary sites within the 3′ untranslated regions (UTRs) of their messenger RNA (mRNA) targets (Reinhart et al., 2000; Ambros, 2004; Bartel, 2009). Nowadays, a large number of experimental studies have proved that miRNAs regulate multiple biological activities and per miRNA can regulate hundreds of gene targets (Lee et al., 1993; Pasquinelli and Ruvkun, 2002; Brennecke et al., 2003; Lin et al., 2003; Cheng et al., 2005; Karp and Ambros, 2005; Miska, 2005; Pillai et al., 2005; Cui et al., 2006; Lu et al., 2008; Bartel, 2009; Alshalalfa and Alhajj, 2013). Moreover, miRNAs have potential influences on almost all genetic pathways, and the upregulation and downregulation of miRNA expression in the human body are correlated to various complex diseases (Liu et al., 2008). It indicates that miRNAs have close associations with many complex diseases, and miRNAs may be used as a tumor suppressor gene to treat cancer in clinical medicine (Cheng et al., 2005). For example, the abnormal expression of miR-21 could be conducive to the growth and spread of human hepatocellular cancer (HCC) via the regulation of phosphatase and tensin homolog (PTEN) expression and PTEN-dependent pathways (Meng et al., 2007). MiR-10b is expressed in metastatic breast cancer cells highly and has a positive regulatory effect on cell migration and invasion (Ma et al., 2007). Research further suggested that the overexpression of miR-17-92 in lung cancer could enhance cell proliferation (Hayashita et al., 2005). Moreover, the miRNA family of let-7 was reported to downregulate in lung cancers and regulate an oncogene of RAS, so the inhibition of let-7 may help in the treatment of the cancer (Johnson et al., 2005). Also, through targeting an antiapoptotic factor of B-cell lymphoma-2 (BCL2), miR-15 and miRNA-16 were proved to downregulate in chronic lymphocytic leukemias and induce apoptosis (Cimmino et al., 2005). Certainly, identification of potential miRNA–disease associations has become a very significant research goal in the field of biomedical research. Predicting potential miRNAs related to diseases would promote people’s understanding of the pathogenesis of diseases at the molecular level and benefit for the diagnosis, treatment, and prevention of diseases. Recently, some reliable databases have been developed to store experimental verified miRNA–disease associations, such as HMDD v2.0 (Li et al., 2014), miR2Disease (Jiang et al., 2009), and dbDEMC (Yang et al., 2010). Using traditional experiment approach to identify potential miRNA-disease associations is usually complex, time consuming and expensive. It is an urgent need for scholars to develop calculation models to predict new miRNA–disease associations. We expect that miRNA–disease pairs with high scores could be selected for experimental verification, which would significantly reduce the time and cost of biological experiments.

Great progress has been made in developing calculation models for the potential miRNA–disease association prediction in recent years. These prediction models are usually proposed by the consideration of complex network-based or machine learning-based methods (Chen et al., 2019a). For the experimentally confirmed miRNA–disease associations that have been collected, a lot of calculation models were put forward for the identification of new miRNA–disease associations on the basis of the hypothesis that functionally similar miRNAs are often associated with phenotypically similar diseases (Perez-Iratxeta et al., 2002; Aerts et al., 2006). In 2013, human disease-related miRNA prediction (HDMP), an effective prediction algorithm based on weighted *k* most similar neighbors, was proposed by Xuan et al. (2015). In the model, functional similarity between each miRNA pair was calculated by combining the information of their related disease terms and disease phenotype similarity. Then the possibility of unobserved miRNA–disease pairs was predicted via the sum of subscores of miRNA’s *k* neighbor. The subscore for a neighbor of a miRNA can be calculated based on the weight of the neighbor and the functional similarity between the neighbor and the miRNA. In 2014, based on known miRNA–disease associations, disease similarity, and miRNA similarity, a global method of regularized least squares for miRNA–disease association (RLSMDA) was introduced by Chen and Yan (2014) to uncover novel associations between miRNAs and diseases under the framework of a semisupervised classifier. In 2015, based on the constructed miRNA functional network, another new model of miRNAs associated with diseases prediction (MIDP) was developed by Xuan et al. (2015) to prioritize candidate miRNAs for investigated diseases with known related miRNAs. In the model, for the marked nodes and unmarked nodes, transition matrices are different, and the transition weight of marked nodes was higher than that of unmarked nodes. Moreover, due to the fact that MIDP could not predict potential miRNAs (diseases) associated with new diseases (miRNAs) without any known related miRNAs (diseases), an extension approach of MIDPE was also proposed to predict potential miRNAs (diseases) associated with new diseases (miRNAs). Chen et al. (2017) published a model of ranking-based KNN for miRNA–disease association prediction (RKNNMDA), in which the KNN approach was employed to gain the *k*-nearest-neighbors of each miRNA and disease according to the collected data information. Then, based on the Hamming loss of per disease pair and miRNA pair, a support vector machine (SVM) ranking model was introduced to achieve scores of potential miRNA–disease associations. Furthermore, Chen and Huang (2017) presented a computational model named Laplacian regularized sparse subspace learning for miRNA–disease association prediction (LRSSLMDA), which projected miRNAs’ feature and diseases’ feature into a common subspace. Then, the local structures of the training data were obtained based on Laplacian regularization, and the final predicted scores would be obtained by carrying out the L1-norm constraint. Chen et al. (2018a) put forward a machine learning-based method of extreme gradient boosting machine for miRNA–disease association prediction (EGBMMDA), in which a feature vector for the miRNA–disease pair was established by merging three matrices of miRNA functional similarity, disease semantic similarity, and known miRNA–disease associations. Then, based on the characteristics and the gradient boosting framework, a regression tree was applied to obtained scores of potential miRNA–disease associations. In the same year, a computational model of ensemble learning and link prediction for miRNA–disease association prediction (ELLPMDA) was brought forward by Chen et al. (2018f); they inferred new miRNA–disease associations via the weight-based integration of three classified results gained from common neighbors, Jaccard index and Katz index. Also, from the angle of reducing the noise of the original collected known miRNA–disease association information, Chen et al. (2018e) further brought up a calculation method of matrix decomposition and heterogeneous graph inference for miRNA–disease association prediction (MDHGI). The sparse learning method was carried out firstly on the initial association information to reduce noise. Then, an iterative formula for propagating miRNA and disease information was established based on the built heterogeneous network to predict potential miRNA–disease associations. Besides, Chen et al. (2018c) presented a novel method of inductive matrix completion for miRNA–disease association prediction (IMCMDA) through enforcing a low-rank inductive matrix completion approach on the collected datasets. Chen et al. (2018d) also developed a prediction model of bipartite network projection for miRNA–disease association prediction (BNPMDA). In the model, the bias ratings for miRNAs and diseases were built based on agglomerative hierarchical clustering. Then, through assigning transfer weights to resource allocation links between miRNAs and diseases according to the bias ratings, the bipartite network recommendation algorithm was implemented to predict the potential miRNA–disease associations. Chen et al. (2019b) put forward a machine learning-based method named ensemble of decision tree-based miRNA–disease association prediction (EDTMDA), which identifies potential disease–miRNA association by implementing ensemble learning based on decision trees (DTs) and dimensionality reduction based on principal component analysis (PCA). In recent years, Chen et al. (2021) further proposed the neighborhood constraint matrix completion for miRNA–disease association prediction (NCMCMDA), which combined the neighborhood constraint with matrix completion. The prediction problem in NCMCMDA can be transformed into an optimization problem, and a fast iterative shrinkage–thresholding algorithm was implemented to solve it.

Some scholars have also introduced some calculation models on the basis of various types of association networks, rather than limited to the miRNA–disease network. In 2014, through the analysis of miRNA–protein associations and protein–disease associations, Mork et al. (2014) developed a scoring scheme for the potential miRNA–disease association prediction. In 2016, through taking advantage of miRNA–disease associations, miRNA–neighbor associations, miRNA–target associations, miRNA–word associations, and miRNA–family associations, Pasquier and Gardes (2016) expressed the distribution information of miRNAs and diseases in a high-dimensional vector space and then inferred association scores between miRNAs and diseases according to their vector similarity. In 2017, based on the phenome–miRNA network constructed by known miRNA–disease associations, miRNA functional similarity, disease semantic similarity, and phenotypic similarity, a combinatorial prioritization algorithm was proposed by Yu et al. (2017) to predict potential miRNA–disease associations. In 2018, through constructing a three-layer heterogeneous network based on the integration of known miRNA–lncRNA interactions, miRNA–disease associations, miRNA similarity, disease similarity, and lncRNA similarity, Chen et al. (2018b) designed a method of triple-layer heterogeneous network-based inference for miRNA–disease association prediction (TLHNMDA) by establishing two information spreading iterative formulas.

In this manuscript, based on a multilayer heterogeneous network established by known miRNA–disease associations, disease semantic similarity, miRNA functional similarity, and Gaussian interaction profile kernel similarity for diseases and miRNAs, we put forward a calculating model of biased random walk with restart on multilayer heterogeneous networks for miRNA–disease association prediction (BRWRMHMDA). In the model, degree-based biased random walk with restart (BRWR) was implemented to predict potential miRNA–disease associations on the basis of the constructed multilayer heterogeneous network. For evaluating the property of the introduced calculation model, local leave-one-out cross-validation (LOOCV) was presented and the outcome showed that BRWRMHMDA possesses an AUC of 0.8310 in local LOOCV. In the case study, we not only employed BRWRMHMDA to infer candidate miRNAs for esophageal neoplasms in the light of known miRNA–disease associations extracted from HMDD v2.0 (Li et al., 2014) but also implemented the model to predict breast neoplasms-associated miRNAs on the basis of known miRNA–disease associations collected from HMDAD v1.0. From the result of LOOCV and the case study, we can be sure that BRWRMHMDA has better prediction ability, and BRWRMHMDA can be used to predict potential miRNA–disease associations.

## Materials and Methods

### Human miRNA–Disease Association

The dataset of 5,430 experimentally verified associations between 383 diseases and 495 miRNAs came from the HMDD v2.0 database (Li et al., 2014). We used the variables **nm** and **nd** to refer to the number of diseases and miRNAs in the dataset, respectively. Afterward, an adjacency matrix *A* was established to indicate known miRNA–disease associations. If miRNA *m*(*i*) is related to *d*(*j*), the value of entity *A*(*i*,*j*) would equal to 1, otherwise 0.

(1) $$A\,\left(i,j\right)=\left\{ \begin{array}{c} 1,i\,f\,m\,i\,R\,N\,A\,m\,\left(j\right)\,i\,s\,r\,e\,l\,a\,t\,e\,d\,t\,o\,d\,i\,s\,e\,a\,s\,e\,d\,\left(i\right)\\ 0,o\,t\,h\,e\,r\,w\,i\,s\,e \end{array}\right.$$

### MiRNA Functional Similarity

Since functionally similar miRNAs are more likely to be associated with phenotypically similar diseases on the basis of the previous study (Wang et al., 2010), we got the information of miRNA functional similarity from http://www.cuilab.cn/files/images/cuilab/misim.zip. After that, we constructed a miRNA functional similarity matrix *FS* with the row and column of **nm**. It is remarkable that the value of entity *FS*(*i*,*j*) refers to the similarity score between miRNA *m*(*i*) and miRNA *m*(*j*).

### Disease Semantic Similarity Model 1

Each disease can be described as a directed acyclic graph (DAG) according to previous literature (Wang et al., 2010). For example, disease *D* can be described as *DAG* = (*D*,*T*(*D*),*E*(*D*)), where *T*(*D*) refers to all disease nodes, and *E*(*D*) indicates all edges that connect disease nodes based on *DAG*(*D*). Inspired by previous work (Xuan et al., 2013), the contribution value of disease *d* in *DAG*(*D*) to the semantic value of disease *D* can be defined as follows:

(2) $$\left\{ \begin{array}{c} D_{D}\,1\,\left(d\right)=1\;i\,f\,d=D\\ D_{D}\,1\,\left(d\right)=\max\left\{\Delta^{*}\,D_{D}\,1\,\left(d^{\prime }\right)|d^{\prime }\in c\,h\,i\,l\,d\,r\,e\,n\,o\,f\,d\right\}\;i\,f\,d\neq D \end{array}\right.$$

where Δ is the semantic contribution decay factor, and the semantic value of disease *D* can be described as follows:

(3) $$D\,V\,1\,\left(D\right)=\sum_{d\in T\,\left(D\right)}D_{D}\,1\,\left(d\right)$$

Considering that two diseases would have greater similarity if they share larger part of their *DAGs*, we defined the semantic similarity between disease *d(i)* and *d(j)* in disease semantic similarity model 1 as follows:

(4) $$\mathrm{SS1}\,\left(d\,\left(i\right),d\,\left(j\right)\right)=\frac{\sum_{t\in T\,\left(d\,\left(i\right)\right)\cap T\,\left(d\,\left(j\right)\right)}\left(D_{d\,\left(i\right)}\,1\,\left(t\right)+D_{d\,\left(j\right)}\,1\,\left(t\right)\right)}{D\,V\,1\,\left(d\,\left(i\right)\right)+D\,V\,1\,\left(d\,\left(j\right)\right)}$$

### Disease Semantic Similarity Model 2

Also inspired by previous work (Xuan et al., 2013), we also introduced disease semantic similarity model 2. For two diseases in the same layer of *DAG*(*D*), if the first disease occurs more frequently in *DAG*(*D*) than the second disease, the second disease would be regarded to be more specific to disease *D*. By the consideration of the idea that the contribution of different disease terms in the same layer of *DAG*(*D*) may be the difference, the contribution of disease *d* in *DAG*(*D*) to the semantic value of disease *D* could be described as follows:

(5) $$D_{D}\,2\,\left(d\right)=-\log\left[\frac{t\,h\,e\,n\,u\,m\,b\,e\,r\,o\,f\,D\,A\,G\,s\,i\,n\,c\,l\,u\,d\,i\,n\,g\,d}{t\,h\,e\,n\,u\,m\,b\,e\,r\,o\,f\,d\,i\,s\,e\,a\,s\,e}\right]$$

The value of semantic similarity in disease semantic similarity model 2 between disease *d*(*i*) and *d*(*j*) could be calculated as follows:

(6) $$\mathrm{SS2}\,\left(d\,\left(i\right),d\,\left(j\right)\right)=\frac{\sum_{t\in T\,\left(d\,\left(i\right)\right)\cap T\,\left(d\,\left(j\right)\right)}\left(D_{d\,\left(i\right)}\,2\,\left(t\right)+D_{d\,\left(j\right)}\,2\,\left(t\right)\right)}{D\,V\,2\,\left(d\,\left(i\right)\right)+D\,V\,2\,\left(d\,\left(j\right)\right)}$$

where

(7) $$D\,V\,2\,\left(D\right)=\sum_{d\in T\,\left(D\right)}D_{D}\,2\,\left(d\right)$$

### Gaussian Interaction Profile Kernel Similarity

The calculation of Gaussian interaction profile kernel similarity for diseases and miRNAs depends on the topologic information of known miRNA–disease associations (van Laarhoven et al., 2011). For diseases, we used a binary vector *IP*(*d*(*u*)) (i.e., the *u*th row of the adjacency matrix *A*) to indicate the interaction profiles of disease *d*(*u*). Accordingly, the Gaussian interaction profile kernel similarity between diseases *d*(*u*) and *d*(*v*) can be described.

(8) $$K\,D\,\left(d\,\left(u\right),d\,\left(v\right)\right)=\exp\left(-\gamma_{d}\,{||I\,P\,\left(d\,\left(u\right)\right)-I\,P\,\left(d\,\left(v\right)\right)||}^{2}\right)$$

The parameter *γ_*d*_* was used to regulate the kernel bandwidth and could be acquired via the normalization of a new bandwidth *$\gamma_{d}^{\prime }$* by the average number of associated miRNAs for each disease.

(9) $$\gamma_{\text{d}}=\gamma_{d}^{\prime }/\left(\frac{1}{n\,d}\,\sum_{u=1}^{n\,d}{||I\,P\,\left(d\,\left(u\right)\right)||}^{2}\right)$$

For miRNAs, the binary vector **IP*(*m*(*i*))* (i.e., the *i*th column of the adjacency matrix *A*) was introduced to indicate the interaction profiles of miRNA **m*(*i*).* At last, the Gaussian interaction profile kernel similarity between miRNA **m*(*i*)* and **m*(*j*)* can be constructed as follows:

(10) $$K\,M\,\left(m\,\left(i\right),m\,\left(j\right)\right)=\exp\left(-\gamma_{m}\,{||I\,P\,\left(m\,\left(i\right)\right)-I\,P\,\left(m\,\left(j\right)\right)||}^{2}\right)$$

(11) $$\gamma_{\text{m}}=\gamma_{m}^{\prime }/\left(\frac{1}{n\,m}\,\sum_{i=1}^{n\,m}{||I\,P\,\left(m\,\left(i\right)\right)||}^{2}\right)$$

### Integrated Similarity for miRNAs and Diseases

Based on past work (Chen et al., 2016), integrated similarity for a pair of diseases (**d*(*u*)* and **d*(*v*)*) can be defined via the combination of disease semantic similarity and Gaussian interaction profile kernel similarity for diseases. The formula of integrated similarity for diseases is displayed as follows:

(12) $$\begin{array}{cc} S\,D\,\left(d\,\left(u\right),d\,\left(v\right)\right)= & \\ \left\{ \begin{array}{c} \frac{S\,S\,1\,\left(d\,\left(u\right)+d\,\left(v\right)\right)+S\,S\,2\,\left(d\,\left(u\right),d\,\left(v\right)\right)}{2}\;d\,\left(u\right)\,a\,n\,d\,d\,\left(v\right)\,h\,a\,s\\ s\,e\,m\,a\,n\,t\,i\,c\,s\,i\,m\,i\,l\,a\,r\,i\,t\,y\\ K\,D\,\left(m\,\left(u\right),m\,\left(v\right)\right)\;o\,t\,h\,e\,r\,w\,i\,s\,e\; \end{array}\right. & \end{array}$$

Also, the integrated similarity for a pair of miRNAs (**m*(*i*)* and **m*(*j*))* could be formed by taking miRNA functional similarity with Gaussian interaction profile kernel similarity for miRNA into account (Chen et al., 2016).

(13) $$\begin{array}{c} S\,M\,\left(m\,\left(i\right),m\,\left(j\right)\right)=\\ \left\{ \begin{array}{c} F\,S\,\left(m\,\left(i\right),m\,\left(j\right)\right)\;m\,\left(i\right)\,a\,n\,d\,m\,\left(j\right)\,h\,a\,s\,f\,u\,n\,c\,t\,i\,o\,n\,a\,l\,s\,i\,m\,i\,l\,a\,r\,i\,t\,y\\ K\,M\,\left(m\,\left(i\right),m\,\left(j\right)\right)\;o\,t\,h\,e\,r\,w\,i\,s\,e\; \end{array}\right. \end{array}$$

### BRWRMHMDA

**FIGURE 1:**
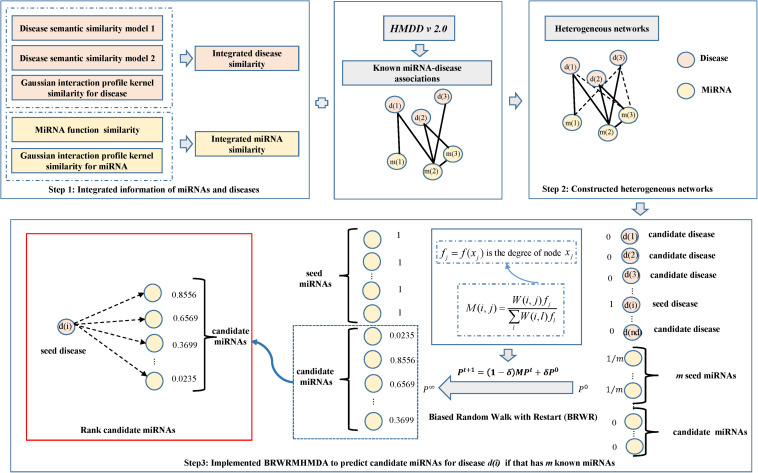
Comparing to the other calculating algorithms (ELLPMDA, IMCMDA, EGBMMDA, MDHGI, TLHNMDA, MaxFlow, RLSMDA, HDMP, WBSMDA, MirAI, and MIDP) in terms of AUCs, BRWRMHMDA gained a better AUC value of 0.8310. It indicates that the proposed model is more suitable for the miRNA–disease association prediction.

Via the integration of known miRNA–disease associations, disease semantic similarity, miRNA functional similarity, and Gaussian interaction profile kernel similarity for miRNAs and diseases, we put forward a calculating model of BRWRMHMDA based on the degree for the identification of potential miRNA–disease associations by enforcing BRWR on a constructed multilayer heterogeneous network according to previous work (Bonaventura et al., 2014) (see Figure 1).

In the model, based on the constructed multisource dataset, we used *W*_*dd*_, *W*_*mm*_,*W*_*dm*_ to represent the initial matrix of integrated disease similarity, integrated miRNA similarity, and known miRNA–disease associations, respectively. Then, the multilayer heterogeneous network was constructed and described as $W\text{=}\left[\begin{array}{cc} W_{dd} & W_{dm}\\ W_{md} & W_{mm} \end{array}\right]$. In BRWR, if we predicted potential miRNAs for disease *d*(*i*), the disease *d*(*i*) is the seed node in the disease network. If the miRNA *m*(*j*) is associated with disease *d*(*i*), miRNA *m*(*j*) is the seed node for disease *d*(*i*) in the miRNA network. If the miRNA *m*(*j*) has no known association with disease *d*(*i*), miRNA *m*(*j*) is the candidate node for disease *d*(*i*) in the miRNA network. For predicting potential miRNAs for disease *d*(*i*), the original probability vector *v*_0_ of the miRNA network is computed through assigning equal probability to the seed nodes in the miRNA network with a total equal to 1. In the disease network, the probability value of 1 was assigned to *d*(*i*), and the probability value of 0 was assigned to other diseases to form *u*_0_, where the initial seed node probability $P^{0}=\left[\begin{array}{c} {\text{α}}^{*}u_{0}\\ {\left(1-\text{α}\right)}^{*}v_{0} \end{array}\right]$; α and (1−α) refer to the weight of the disease network and the miRNA network, respectively.

Seed nodes at each step move to their immediate neighbors with a probability (1−δ) or return to the seed nodes with a restart probability δ (δ ∈ (0,1)). *P*^0^ was the initial probability vector, and *P*^*t* + 1^ was a probability vector of node at time **t* + 1*, which could be defined as follows:

(14) $$P^{t+1}=\left(1-\delta \right)\,M\,P^{t}+\delta \,P^{0}$$

where matrix $M\text{=}\left[\begin{array}{cc} M_{dd} & M_{dm}\\ M_{md} & M_{mm} \end{array}\right]$ is the transition matrix of our established network. In random walk with restart (RWR), the transition probability *M*(*i*,*j*) of a walker from node **i** to node **j** can be described as follows:

(15) $$M\,\left(i,j\right)=\frac{W\,\left(i,j\right)}{\sum_{l}W\,\left(i,l\right)}$$

where *W*(*i*,*j*) is the similarity between node *i* and node *j*. In this model, BRWR of degree biased random walk was proposed to identify potential miRNA–disease associations. Biases were usually considered to be related to graph topological properties. For example, a walk at node *x*_*i*_ selects it neighbors of *x*_*j*_ with a probability *f*_*j*_ = *f*(*x*_*j*_) relying on the node property *x*_*j*_. Usually, the node property can be described as a function of the vertex properties (the network degree, closeness centrality, etc.) or the edge properties (multiplicity or shortest path), or the combination of them (Gomez-Gardenes and Latora, 2008). There are other related bias choice of maximal entropy (Burda et al., 2009). Thus, the transition probability of a walker from **i** to **j** in BRWR can be defined as

(16) $$M\,\left(i,j\right)=\frac{W\,\left(i,j\right)\,f_{j}}{\sum_{l}W\,\left(i,l\right)\,f_{l}}$$

Therefore, in the disease similarity network, the transition probability from vertex *d*_*i*_ to *d*_*j*_ can be defined as

(17) $$\begin{array}{c} M_{d\,d}\,\left(i,j\right)=p\,\left(d_{j}|d_{i}\right)=\\ \left\{ \begin{array}{c} W_{d\,d}\,\left(i,j\right)\,f_{j}/\sum_{j}W_{d\,d}\,\left(i,j\right)\,f_{j}\;i\,f\,\sum_{j}W_{d\,m}\,\left(i,j\right)=0\\ \left(1-\lambda \right)\,W_{d\,d}\,\left(i,j\right)\,f_{j}/\sum_{j}W_{d\,d}\,\left(i,j\right)\,f_{j}\;o\,t\,h\,e\,r\,w\,i\,s\,e\; \end{array}\right. \end{array}$$

In the miRNA similarity network, the transition probability from *m*_*i*_ to*m*_*j*_ can be defined as

(18) $$\begin{array}{c} M_{m\,m}\,\left(i,j\right)=p\,\left(m_{j}|m_{i}\right)=\\ \left\{ \begin{array}{c} W_{m\,m}\,\left(i,j\right)\,f_{j}/\sum_{j}W_{m\,m}\,\left(i,j\right)\,f_{j}\;i\,f\,\sum_{j}W_{d\,m}\,\left(j,i\right)=0\\ \left(1-\lambda \right)\,W_{m\,m}\,\left(i,j\right)\,f_{j}/\sum_{j}W_{m\,m}\,\left(i,j\right)\,f_{j}\;o\,t\,h\,e\,r\,w\,i\,s\,e\; \end{array}\right. \end{array}$$

In the miRNA–disease association network, the transition probability from vertex *d*_*i*_ to *m*_*j*_ can be defined as

(19) $$\begin{array}{c} M_{d\,m}\,\left(i,j\right)=p\,\left(m_{j}|d_{i}\right)=\\ \left\{ \begin{array}{c} \lambda \,W_{d\,m}\,\left(i,j\right)\,f_{j}/\sum_{j}W_{d\,m}\,\left(i,j\right)\,f_{j}\;i\,f\,\sum_{j}W_{d\,m}\,\left(i,j\right)\neq 0\\ 0\;o\,t\,h\,e\,r\,w\,i\,s\,e \end{array}\right. \end{array}$$

The transition probability from vertex *m*_*i*_ to *d*_*j*_ can be defined as

(20) $$\begin{array}{c} M_{m\,d}\,\left(i,j\right)=p\,\left(d_{j}|m_{i}\right)=\\ \left\{ \begin{array}{c} \lambda \,W_{d\,m}\,\left(j,i\right)\,f_{j}/\sum_{j}W_{d\,m}\,\left(j,i\right)\,f_{j}\;i\,f\,\sum_{j}W_{d\,m}\,\left(j,i\right)\neq 0\\ 0\;o\,t\,h\,e\,r\,w\,i\,s\,e \end{array}\right. \end{array}$$

In this paper, we focus on the case of BRWR by considering the degree nodes. Therefore, *f*_*j*_ = *f*(*x*_*j*_) in the model is the degree of node *x*_*j*_ in the transition probability. The degree **f*_*i*_* of a disease node **i** is defined by computing the number of edges involved in the disease node. Therefore, in the disease similarity network, the degree of disease node *j* can be defined as *f*_*j*_ = ∑_*i*_*W*_*dd*_(*i*,*j*). In the miRNA similarity network, the degree of miRNA node *j* can be defined as *f*_*j*_ = ∑_*i*_*W*_*mm*_(*i*,*j*). In the transition probability matrix of the miRNA–disease association network, the degree of miRNA *m*_*j*_ can be described as *f*_*j*_ = ∑_*i*_*W*_*dm*_(*i*,*j*). Also, in the transition probability matrix of the miRNA–disease association network, the degree of disease *d*_*j*_ can be described as *f*_*j*_ = ∑_*i*_*W*_*dm*_(*j*,*i*). Therefore, based on BRWR of degree nodes, the potential miRNA–disease associations would be obtained.

## Results

### Performance Evaluation

**FIGURE 2:**
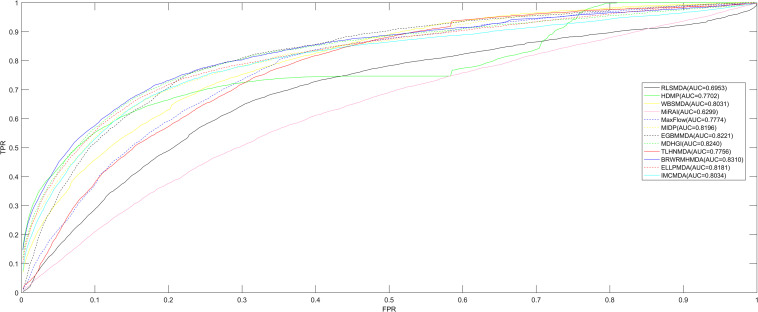
Flowchart of BRWRMHMDA to prioritize candidate miRNAs for diseases. Through employing BRWR on the established heterogeneous networks, final scores *p*^∞^ of candidate miRNAs predicted for each disease would be gotten after some steps.

Since BRWR is a local calculating method, it cannot infer candidate miRNAs for all diseases simultaneously. Therefore, in order to analyze the performance of BRWRMHMDA, the proposed method has been extensively compared with some classic algorithms (ELLPMDA, IMCMDA, EGBMMDA, MDHGI, TLHNMDA, MaxFlow, RLSMDA, HDMP, WBSMDA, MirAI, and MIDP) based on the 5,430 known miRNA–disease associations from the HMDD v2.0 database (Li et al., 2014) via local LOOCV. In local LOOCV, each known miRNA–disease association was considered as a test sample in turn, and the rest of 5,429 known associations were treated as training samples. After enforcing BRWRMHMDA, the score of the test sample would be sorted with the scores of all unobserved pairs between miRNAs and the investigated disease. The proposed approach would be regarded as reliable if the test sample’s ranking is higher than a set threshold. Then a receiver operating characteristics (ROC) curve with the true positive rate (TPR, sensitivity) versus the false positive rate (FPR, 1-specificity) at various thresholds would be drawn. Sensitivity refers to the percentage of test samples ranked higher than the given threshold, and specificity refers to the percentage of candidates ranked lower than the threshold. Finally, the area under the ROC curve (AUC) was calculated to accurately evaluate the prediction ability of BRWRMHMDA. The value of the AUC is between 0 and 1, and the higher the value of the AUC, the better the prediction performance of the algorithm. If the value of the AUC is 0.5, the prediction performance of BRWRMHMDA is random. The final assessment results showed that BRWRMHMDA has better prediction performance with an AUC of 0.8310 than those of the other server classical algorithms of ELLPMDA (0.8181), IMCMDA (0.8034), EGBMMDA (0.8221), MDHGI (0.8240), TLHNMDA (0.7756), MaxFlow (0.7774), RLSMDA (0.6953), HDMP (0.7702), WBSMDA (0.8031), MirAI (0.6299), and MIDP (0.8196) (see Figure 2). Here, the AUC value of MirAI is lower than that reported in its literature (Pasquier and Gardes, 2016) because MirAI was proposed on the basis of a collaborative filtering algorithm affected by the data sparsity problem. Compared with the training set in the original literature, our dataset is relatively scarce. The training set in the original literature contains 83 diseases and at least 20 known related miRNAs for each disease, while our training set contains 383 diseases and most diseases-related miRNAs are rare.

### Case Studies

In order to further analyze the performance of the algorithm effectively, we carried out two types of case studies. The first type of case studies is the prediction of potential miRNAs associated with esophageal neoplasms based on the known miRNA–disease association collect from HMDD v2.0. The second type of case studies is the prediction of potential miRNAs associated with breast neoplasms based on the known miRNA–disease association collect from HMDD v1.0.

Esophageal neoplasm is one of the most lethal cancers in the world; its main nature is highly invasive and of low survival rate (Domper Arnal et al., 2015). The disease contains two main histological types of squamous cell cancer and adenocarcinoma (Zhang et al., 2016). Malnutrition is a main risk factor for esophageal squamous cell carcinoma (ESCC), and obesity is the main risk factor for esophageal adenocarcinoma (Domper Arnal et al., 2015). Accordingly, looking for sensitive molecular biomarkers and individual treatment approach for early diagnosis of esophageal cancer has become the main clinical and basic research direction. Numerous studies suggested that miRNAs play an important role in diseases and can be a biomarker for esophageal neoplasms’ treatment. For example, miR-506 was abnormally expressed in a variety of tumors and could be used as a prognostic biomarker for ESCC (Li et al., 2016). Besides, plasma miR-718 was reported to downregulate in ESCC patients and might be treated as a potential diagnostic marker for the disease (Sun et al., 2016). Here, we employed BRWRMHMDA to prioritize candidate miRNAs for esophageal neoplasms according to the dataset of 5,430 known miRNA–disease associations between 383 diseases and 495 miRNAs. As a result, of the first 50 miRNAs predicted for esophageal neoplasms in the ranking, 49 miRNAs have been confirmed by the database of dbDEMC and miR2Disease (see Table 1). For example, the predicted association score between hsa-mir-125b and esophageal neoplasms is ranked first. Yu et al. (2020) have found that hsa-mir-125b suppresses cell proliferation and metastasis by targeting HAX-1 in ESCC, which proves that hsa-mir-125b is related to esophageal neoplasms. Moreover, the predicted association score between hsa-mir-200b and esophageal neoplasms is ranked second. Researchers have confirmed that hsa-mir-200b is downregulated in ESCC in the comparison of the respective adjacent benign tissues (Zhang et al., 2014). Therefore, hsa-mir-200b is associated with esophageal neoplasms.

**TABLE 1:** The implementation of BRWRMHMDA to prioritize candidate miRNAs for esophageal neoplasms based on experimentally confined miRNA–disease associations collected from HMDD v2.0 and 47 of the first 50 predicted miRNAs were confirmed.

miRNA	Evidence	miRNA	Evidence
hsa-mir-125b	dbDEMC	hsa-mir-429	dbDEMC
hsa-mir-200b	dbDEMC	hsa-mir-106a	dbDEMC
hsa-mir-18a	dbDEMC	hsa-mir-24	dbDEMC
hsa-mir-17	dbDEMC	hsa-mir-30c	dbDEMC
hsa-mir-221	dbDEMC	hsa-mir-218	unconfirmed
hsa-mir-19b	dbDEMC	hsa-mir-93	dbDEMC
hsa-mir-16	dbDEMC	hsa-mir-132	dbDEMC
hsa-mir-1	dbDEMC	hsa-mir-30a	dbDEMC
hsa-mir-222	dbDEMC	hsa-mir-127	dbDEMC
hsa-let-7i	dbDEMC	hsa-mir-195	dbDEMC
hsa-mir-29a	dbDEMC	hsa-mir-199b	dbDEMC
hsa-let-7e	dbDEMC	hsa-mir-10b	dbDEMC
hsa-let-7d	dbDEMC	hsa-mir-15b	dbDEMC
hsa-mir-29b	dbDEMC	hsa-mir-107	dbdemc and miR2Disease
hsa-let-7f	unconfirmed	hsa-mir-7	dbDEMC
hsa-mir-181b	dbDEMC	hsa-mir-224	dbDEMC
hsa-mir-181a	dbDEMC	hsa-mir-18b	dbDEMC
hsa-mir-125a	dbDEMC	hsa-mir-133b	dbDEMC
hsa-let-7g	dbDEMC	hsa-mir-335	dbDEMC
hsa-mir-9	dbDEMC	hsa-mir-194	dbdemc and miR2Disease
hsa-mir-146b	dbDEMC	hsa-mir-302b	dbDEMC
hsa-mir-106b	dbDEMC	hsa-mir-20b	dbDEMC
hsa-mir-182	dbDEMC	hsa-mir-124	dbDEMC
hsa-mir-142	dbDEMC	hsa-mir-373	dbdemc and miR2Disease
hsa-mir-122	unconfirmed	hsa-mir-191	dbDEMC

Breast neoplasm is one of the three most common cancers for women (Siegel et al., 2018). In particular, metastatic breast cancer (MBC) is usually incurable, and about 5% of patients have metastases at diagnosis (Torre et al., 2015). With recent research, miR-10b sponge has been shown to effectively inhibit the growth of MDA-MB-231 and MCF-7 cells in breast cancer (Liang et al., 2016). In addition, miR-223 was demonstrated to function as a potential tumor marker for breast neoplasm through suppressing its protein expression of FOXO1 (Wei et al., 2017). Accordingly, identifying breast neoplasm-related miRNAs is meaningful, which could help the medical diagnosis and treatment for MBC (McGuire et al., 2015). Here, we enforced BRWRMHMDA to infer potential miRNAs related to breast neoplasms on the basis of 1,395 known miRNA–disease associations between 137 diseases and 271 miRNAs collected from HMDD v1.0. The results showed that 48 of the first 50 miRNAs predicted for breast neoplasms have been confirmed by the databases of dbDEMC, miR2Disease, and HMDD v2.0 (see Table 2). For example, hsa-let-7b was predicted to associate with breast neoplasms, and the predicted score is ranked second. It is worth noting that hsa-let-7b can significantly change oncogenic signaling in breast cancer cells. Consequently, hsa-let-7b may have important roles in breast neoplasm progression and can be considered as potential targets for breast neoplasm therapy and diagnosis (Bozgeyik, 2020). Besides, hsa-mir-16 was predicted to be related to breast neoplasms, and the predicted score is ranked third. Haghi et al. indicated that has-mir-16 and has-mir-34a can collaborate in breast tumor suppression, which proved that hsa-mir-16 has association with breast neoplasms.

**TABLE 2:** The implementation of BRWRMHMDA to prioritize candidate miRNAs for breast neoplasms based on experimentally confined miRNA–disease associations collected from HMDD v1.0 and 48 of the first 50 predicted miRNAs were confirmed.

miRNA	Evidence	miRNA	Evidence
hsa-let-7i	dbDEMC and miR2Disease and HMDD	hsa-mir-203	dbDEMC and miR2Disease and HMDD
hsa-let-7b	dbDEMC and HMDD	hsa-mir-32	dbDEMC
hsa-mir-16	dbDEMC and HMDD	hsa-mir-30e	unconfirmed
hsa-let-7e	dbDEMC and HMDD	hsa-mir-532	dbDEMC
hsa-let-7g	dbDEMC and HMDD	hsa-mir-335	dbDEMC and miR2Disease and HMDD
hsa-let-7c	dbDEMC and HMDD	hsa-mir-150	dbDEMC
hsa-mir-92a	HMDD	hsa-mir-199b	dbDEMC and HMDD
hsa-mir-126	dbDEMC and miR2Disease and HMDD	hsa-mir-99a	dbDEMC
hsa-mir-223	dbDEMC and HMDD	hsa-mir-98	dbDEMC and miR2Disease
hsa-mir-92b	dbDEMC	hsa-mir-142	unconfirmed
hsa-mir-373	dbDEMC and miR2Disease and HMDD	hsa-mir-128b	miR2Disease
hsa-mir-101	dbDEMC and miR2Disease and HMDD	hsa-mir-107	dbDEMC and HMDD
hsa-mir-191	dbDEMC and miR2Disease and HMDD	hsa-mir-224	dbDEMC and HMDD
hsa-mir-182	dbDEMC and miR2Disease and HMDD	hsa-mir-27a	dbDEMC and miR2Disease and HMDD
hsa-mir-99b	dbDEMC	hsa-mir-195	dbDEMC and miR2Disease and HMDD
hsa-mir-106a	dbDEMC	hsa-mir-124	dbDEMC and HMDD
hsa-mir-181a	dbDEMC and miR2Disease and HMDD	hsa-mir-30a	miR2Disease and HMDD
hsa-mir-29c	dbDEMC and miR2Disease and HMDD	hsa-mir-520b	dbDEMC and HMDD
hsa-mir-100	dbDEMC and HMDD	hsa-mir-95	dbDEMC
hsa-mir-18b	dbDEMC and HMDD	hsa-mir-23b	dbDEMC and HMDD
hsa-mir-372	dbDEMC	hsa-mir-491	dbDEMC
hsa-mir-24	dbDEMC and HMDD	hsa-mir-183	dbDEMC and HMDD
hsa-mir-130a	dbDEMC	hsa-mir-31	dbDEMC and miR2Disease and HMDD
hsa-mir-15b	dbDEMC	hsa-mir-192	dbDEMC
hsa-mir-196b	dbDEMC	hsa-mir-135a	dbDEMC and HMDD

At last, we have released the whole prediction results via the implementation of BRWRMHMDA for all miRNA–disease pairs between 383 diseases and 495 miRNAs from HMDD v2.0 (see Supplementary Table 1).

## Discussion

Through integrating known miRNA–disease associations, disease semantic similarity, miRNA function similarity, and Gaussian interaction profile kernel similarity for miRNAs and diseases, BRWRMHMDA was employed in this manuscript to prioritize candidate miRNAs for diseases via the implementation of degree-based BRWR on the established networks. The assessment results of LOOCV showed that the developed algorithm outperforms the other 11 classic prediction algorithms in accuracy. We further enforced the proposed algorithm to infer candidate miRNAs for esophageal neoplasms in the light of known miRNA–disease associations extracted from HMDD v2.0 and infer candidate miRNAs for breast neoplasms in the light of known miRNA–disease associations extracted from HMDD v1.0. The results of the case study fully demonstrated the stability of this introduced algorithm. It is worth mentioning that our research group will keep on studying this issue in depth. Furthermore, we hope more external research groups would select potential associations with high prediction scores and verify them based on biological experiment in the future.

Actually, the method’s high accuracy in the miRNA–disease association predictions mainly rely on the following attractive properties. First, the training set of known miRNA–disease associations used in this manuscript was collected from a very reliable database of HMDD v2.0, and the several bioinformatics data (disease semantic similarity, miRNA function similarity, and Gaussian interaction profile kernel similarity for miRNAs and diseases) mentioned in the paper were accurately calculated and integrated. All the reliable biological information mentioned above would attribute to the accuracy of BRWRMHMDA. Second, compared with the machine learning-based methods that randomly select negative samples as the training set, the proposed algorithm only uses positive samples as the training set that would provide higher prediction value. At last, BRWRMHMDA, a degree-biased random walk, could fully take advantage of the information about node degree and improve the prediction accuracy. From the preceding discussion, it is no surprise that this algorithm is superior to other comparison algorithms and has good performance.

However, the proposed model still has some weaknesses and shortcomings. For example, despite the biological information collected here being reliable, the number of 5,430 experimentally verified miRNA–disease associations extracted from HMDD v2.0 is still far from enough. If more associations between miRNAs and diseases are validated, the prediction accuracy of the model would be higher. Moreover, except for the fact that miRNA similarity could be calculated via the consideration of miRNA functional similarity and Gaussian interaction profile kernel for miRNAs, it could also be calculated based on other miRNA features. At the same time, disease similarity could also be calculated based on other disease features. Also, the model could not predict candidate miRNAs for new diseases that have no known related miRNAs. In addition, due to the fact that the proposed algorithm is a local ranking model, it could not infer candidate miRNAs for all diseases simultaneously.

Nowadays, more and more researchers are studying the regulatory interactions between ncRNA classes, as well as the associations between ncRNA and other biological entities including diseases, small molecules, etc. Prediction of ncRNA-related networks will greatly expand our understanding of ncRNA function and its regulatory network. Simultaneously, predictions including miRNA–lncRNA interactions, miRNA–circRNA interactions, drug–target interactions, small molecule–miRNA associations, and disease–lncRNA associations have made great progress. In the field of miRNA–disease association prediction, the number of known miRNA–disease associations is limited, which will affect the prediction performance of the model. In the future, integrating multisource biological data that was mentioned above to build a multilayer heterogeneous network based on machine learning-based method can effectively improve the prediction performance of the model.

## Data Availability Statement

The original contributions presented in the study are included in the article/Supplementary Material, further inquiries can be directed to the corresponding author/s.

## Author Contributions

JQ implemented the experiments, analyzed the result, and wrote the manuscript. C-CW analyzed the result, revised the manuscript, and supervised the project. S-BC and ZM analyzed the result and revised the manuscript. W-DZ and X-LC contributed to the analysis of the data for the manuscript and revised the manuscript. All authors read and approved the final manuscript.

## Conflict of Interest

The authors declare that the research was conducted in the absence of any commercial or financial relationships that could be construed as a potential conflict of interest.

## Publisher’s Note

All claims expressed in this article are solely those of the authors and do not necessarily represent those of their affiliated organizations, or those of the publisher, the editors and the reviewers. Any product that may be evaluated in this article, or claim that may be made by its manufacturer, is not guaranteed or endorsed by the publisher.
